# Genomic stability of mouse spermatogonial stem cells in vitro

**DOI:** 10.1038/s41598-021-03658-1

**Published:** 2021-12-17

**Authors:** Shinichiro Chuma, Mito Kanatsu-Shinohara, Ami Katanaya, Mihoko Hosokawa, Takashi Shinohara

**Affiliations:** 1 Institute for Frontier Life and Medical Sciences, Kyoto University, Kyoto, Japan; 2 Department of Molecular Genetics, Graduate School of Medicine, Kyoto University, Yoshida Konoe, Sakyo-ku, Kyoto, 606-8501 Japan; 3 AMED-CREST, AMED 1-7-1 Otemachi, Chiyodaku, Tokyo 100-0004 Japan

**Keywords:** DNA, Developmental biology, Evolution, Genetics, Stem cells

## Abstract

Germline mutations underlie genetic diversity and species evolution. Previous studies have assessed the theoretical mutation rates and spectra in germ cells mostly by analyzing genetic markers and reporter genes in populations and pedigrees. This study reported the direct measurement of germline mutations by whole-genome sequencing of cultured spermatogonial stem cells in mice, namely germline stem (GS) cells, together with multipotent GS (mGS) cells that spontaneously dedifferentiated from GS cells. GS cells produce functional sperm that can generate offspring by transplantation into seminiferous tubules, whereas mGS cells contribute to germline chimeras by microinjection into blastocysts in a manner similar to embryonic stem cells. The estimated mutation rate of GS and mGS cells was approximately 0.22 × 10^−9^ and 1.0 × 10^−9^ per base per cell population doubling, respectively, indicating that GS cells have a lower mutation rate compared to mGS cells. GS and mGS cells also showed distinct mutation patterns, with C-to-T transition as the most frequent in GS cells and C-to-A transversion as the most predominant in mGS cells. By karyotype analysis, GS cells showed recurrent trisomy of chromosomes 15 and 16, whereas mGS cells frequently exhibited chromosomes 1, 6, 8, and 11 amplifications, suggesting that distinct chromosomal abnormalities confer a selective growth advantage for each cell type in vitro. These data provide the basis for studying germline mutations and a foundation for the future utilization of GS cells for reproductive technology and clinical applications.

## Introduction

Germline cells have a lower mutation frequency than somatic cells^1–4^. The rates and spectra of de novo mutations affect evolutionary speed and direction but also cause genetic diseases. Among many cell types in the germline, spermatogonial stem cells (SSCs) are the only stem cells with self-renewal activity in mammals^5,6^. SSCs maintain spermatogenesis throughout the life of male animals. They continuously divide and produce progenitor cells by self-renewal division, producing an enormous number of progenitor cells. Consequently, the frequency of SSCs in the testis is very low (0.02–0.03% of all germ cells in the testis)^6,7^, making it difficult to distinguish these cells from other committed progenitor cells. In general, stem cells are mitotically quiescent or divide only rarely in most tissues, whereas committed progenitor cells proliferate more actively. Because mutations in stem cells persist and accumulate in the genome, stem cells must have stringent control over their repair machinery. In contrast, progenitor cells divide frequently and differentiate into mature cells. Therefore, these cells do not accumulate mutations in the long term. Thus, it is likely that the mutation rate in SSCs is minimal in the germline lineage.

Although SSC analysis has been hampered by their low frequency, the development of an SSC culture system has allowed the in vitro expansion of SSCs^8^. The addition of glial cell-derived neurotrophic factor (GDNF) and fibroblast growth factor 2 (FGF2), both self-renewal factors for SSCs, stimulated the proliferation of spermatogonia in vitro and induced the formation of grape-like clusters of germ cells. These cells, designated as germline stem (GS) cells, can proliferate for more than 2 years without significant loss of SSC activity to recolonize seminiferous tubules^9^. Upon microinjection into seminiferous tubules, they reinitiate spermatogenesis and produce sperm. By mating with wild-type females, offspring from transplanted GS cells are born in most successful cases. Although these results established that GS cells have SSC activity, they also spontaneously convert into multipotent GS (mGS) cells^10^. These cells do not depend on GDNF or FGF2 but proliferate in the same manner as embryonic stem (ES) cells and contribute to forming germline chimeras by blastocyst injection. Thus, the derivation of two types of stem cells from the male germline created a unique opportunity to analyze a rare SSC population.

Using the culture technique, GS cells were relatively stable in their karyotype and DNA methylation patterns, as reported previously^9,11^. One study has found that GS cells remained euploid and exhibited stable androgenetic DNA methylation patterns after 2 years of culture^9^. They also produced offspring by spermatogonial transplantation. Although androgenetic DNA methylation patterns were maintained after 5 years of culture, one of the two lines showed a partial deletion of chromosome 17^11^. However, they still continued to proliferate despite significantly shortened telomeres. These results contrast with ES and somatic cells. ES cells become aneuploid and exhibit unstable DNA methylation patterns even during short-term cultures^12–14^. In contrast, somatic cells undergo senescence after repeated passages. Although the mechanism of high stability of GS cells is still unknown, it is possible that GS cells have a unique DNA repair machinery. This is suggested by a previous observation that irradiation of GS cells induces apoptosis more easily than ES cells, whereas ES or mGS cells are significantly resistant to the same treatment and continue to proliferate^15^. Although the lower mutation rate in ES cells and its mechanism have been well characterized^16^, little is known about the impact of cell culture on the genome integrity of GS cells.

This study evaluated DNA mutations using whole-genome sequencing (WGS). This technique has revolutionized genetic analysis and contributed to advance the knowledge on genomic stabilities on various stem cell types, including pluripotent stem cells or tissue-specific stem cells. For example, next-generation sequence analysis showed that human ES cells accumulate *TP53* mutations during culture despite their significantly lower mutation rate than somatic cells^17^. This study analyzed two types of GS cell cultures. In the first set of experiments, single GS or mGS cell clones were allowed to expand for 100 population doublings, and DNA mutations were analyzed in two clonally derived cell populations. In the second set of experiments, GS cells were analyzed after 5 years of standard bulk culture condition. The types of mutations and changes in karyotypes were also analyzed.

## Results

### Clonal analyses of genome stability in mouse GS and mGS cells

**Figure 1:**
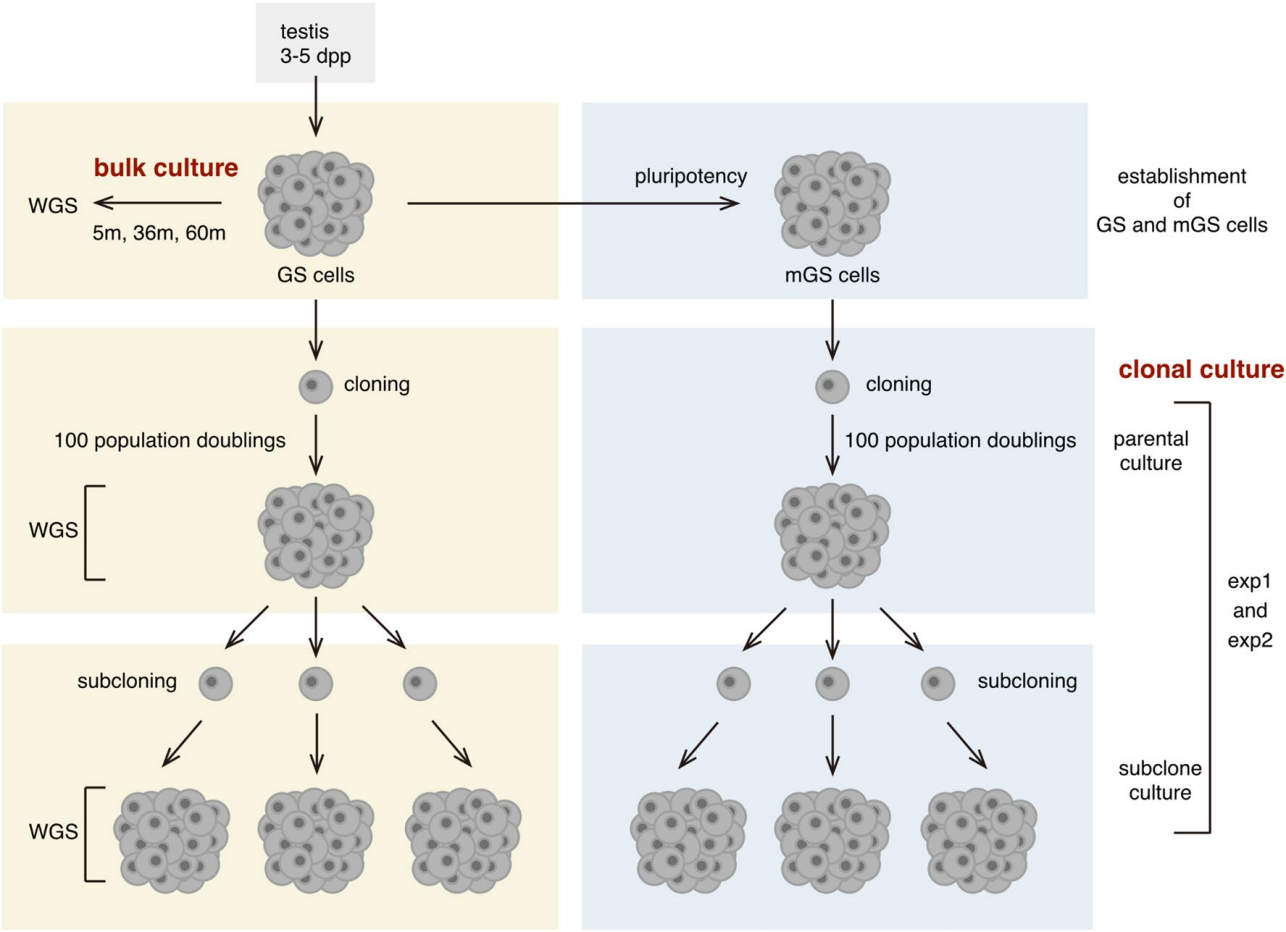
Experimental scheme of clonal mutation analysis of mouse GS and mGS cell lines. GS cells were established from neonatal testicular cells of male mice using MEF cells as a feeder cell layer in the presence of GDNF and FGF2. mGS cells were isolated from GS cell cultures by picking up cell colonies that changed morphology to a pluripotent stem cell-like appearance. mGS cells were maintained on MEF cells under a conventional mouse ES cell culture condition supplemented with leukemia inhibitory factor (LIF) and fetal bovine serum. Clonal parental cultures were derived from single cells of GS and mGS cell lines by plating each cell type at low cell densities and picking up clonally derived colonies, followed by deposition to 96-well plates. These clonal parental cultures were then expanded and maintained for a defined period (100 population doublings) and used for WGS and karyotype analyses. From each clonal parental culture, three subclonal cultures were further derived by recloning and then used for WGS. By comparing WGS data of parental and each subclonal cultures, de novo mutations that accumulated during 100 population doublings were analyzed. Two experimental sets (exp1 and exp2) of these clonal mutation analyses were carried out for GS and mGS cell lines, respectively. GS cell lines were also maintained under a standard bulk culture condition for up to 5 years (5, 36, and 60 months). Mutation accumulation in these standard GS cell cultures was analyzed by comparing WGS data obtained at 5, 36, and 60 months.

To assess the genome stability of mouse GS and mGS cells, de novo mutations that accumulate in clonally derived cell populations of each cell type during a defined culture period were analyzed. To this end, clonal cultures from single cells were first carried out, and each clone of GS and mGS cells was expanded for 100 population doublings (Fig. 1). WGS of these cell populations (parental clonal cultures) represents the genome sequences of single cells that gave rise to clonal cultures, assuming that any cells that acquired specific genetic changes did not dominate the cell population by growth advantage.

From these clonally derived cell populations, three independent subclones were further derived (subclones 1–3) by repeating clonal expansion from single cells. WGS of these subclones represent the genome sequences of single cells that gave rise to subclonal cultures, provided the same assumption, as described above (i.e., no growth advantage of specific cells). By comparative analyses of the genome sequences between “parental clonal cultures” and “subclonal cultures” derived thereof, the number of de novo mutations acquired during the predefined culture period of parental clones (100 population doublings) was estimated. Two independent sets of these clonal culture experiments (exp1 and exp2) were carried out for GS and mGS cells, as summarized in Fig. 1.

### Mutation rates of GS and mGS cell lines

**Figure 2:**
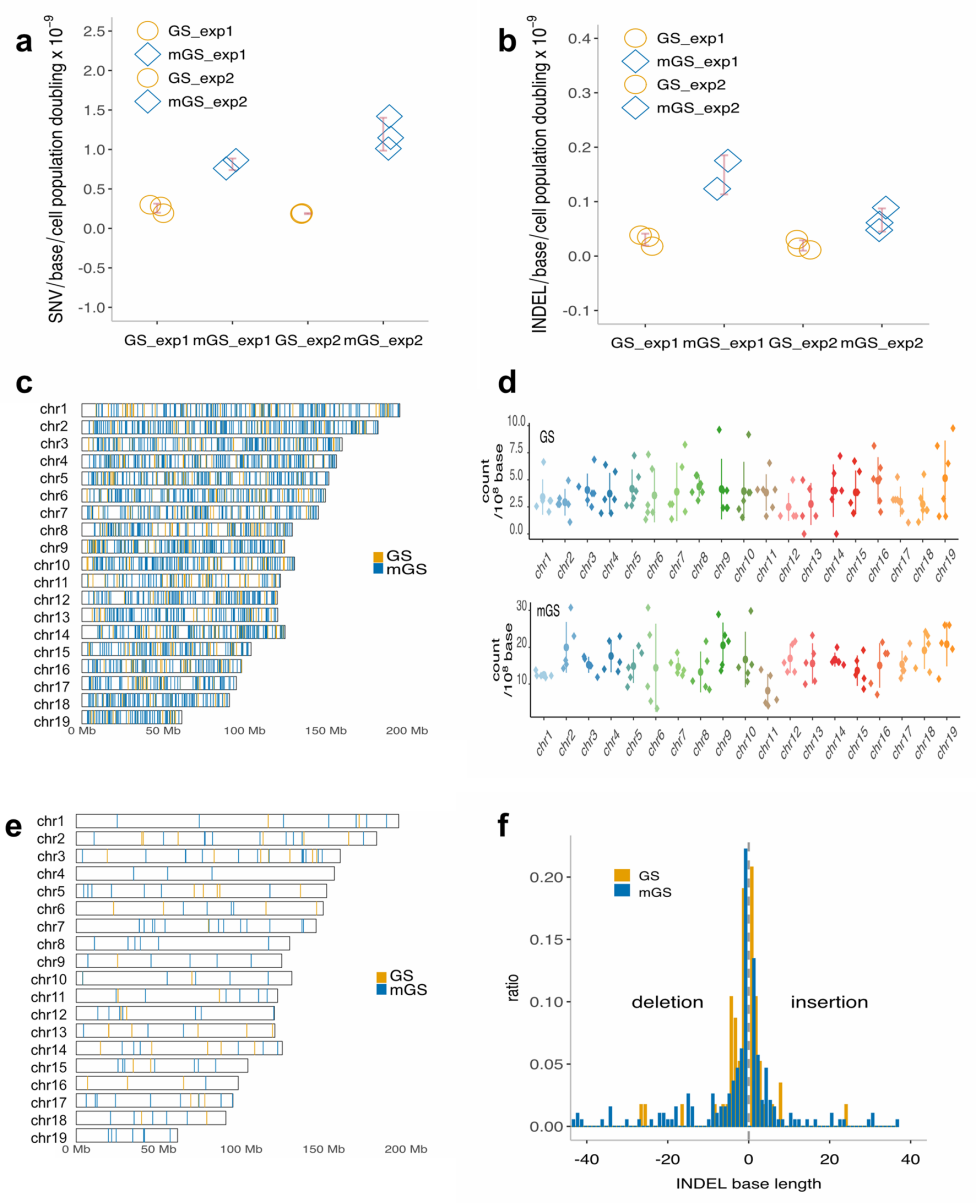
Detection of de novo SNVs and INDELs by WGS of mouse GS and mGS cell lines. (**a** and **b**) Mutation rates of SNVs (**a**) and INDELs (**b**) in GS and mGS cells. The values are the estimated numbers of de novo SNVs (**a**) and INDELs (**b**) detected by the comparative analyses of WGS data of each subclone and corresponding parental cultures of GS and mGS cells divided by the effective diploid genome length in base pairs and by the number of population doublings. To derive these estimated values, a Gauss model was fitted to allele frequency distributions of each sample after trimming de novo mutations with low allele frequencies (see Materials and methods). Data were obtained from two independent experiments (exp1 and exp2). One mGS subclone (subclone2 in exp1) was omitted as an outlier from our analyses. The red bars represent mean ± SD. (**c**) Chromosomal distribution of de novo SNVs in GS and mGS cells. Data from each subclone in exp1 and exp2 were combined for GS cells (orange vertical lines) and mGS cells (blue vertical lines), respectively. The X and Y chromosomes were excluded from our analyses, as the sequence read coverages on these chromosomes were variable due to the abundance of repeat sequences. (**d**) Number of de novo SNVs per chromosome normalized by 100 Mbp. Colored dots represent each subclone of GS (top) and mGS (bottom), with the vertical bars showing mean ± SD. (**e**) Chromosomal distribution of de novo INDELs in GS and mGS cells with the data presented similarly in (**c**). (**f**) Base-pair length of de novo INDELs [insertions on the right (> 0) and deletions on the left (< 0)] of GS cells (orange) and mGS cells (blue), with the data from each subclone in exp1 and exp2 combined.

WGS of parental clones and subclonal cohorts of GS and mGS cells from exp1 and exp2 was carried out using the Illumina sequencing platforms. De novo single nucleotide variants (SNVs) and small insertions and deletions (INDELs) were examined by comparative analyses between parental cultures and corresponding subclonal cohorts. The total estimated numbers of de novo SNVs for GS cells were 84 to 131 for exp1 and 77 to 83 for exp2. To estimate these total numbers of de novo SNVs, de novo SNVs with low allele frequencies were removed, and a Gaussian distribution model was applied (see Materials and methods section). By considering (1) the effective genome region (approximately 2.0 × 10^9^–2.2 × 10^9^ bases), which had more than 10 read coverages in both parental and subclone sequences to ensure high fidelity mutation calling, and (2) population doublings (100), which were used as substitutes for cell division numbers, the derived estimation for the mutation rates of GS cells was 0.18 to 0.29 (mean = 0.22, 95% confidence interval = 0.17–0.28) × 10^−9^ per base per cell generation (Fig. 2a). The number of de novo SNVs estimated for mGS cells was 331 to 370 for exp1 and 429 to 602 for exp2. Subclone 2 of exp1 was removed as an outlier from further analyses, as this subclone exhibited an exceptionally high SNV count (2461) compared to other mGS subclones. The reason for this high SNV count was unknown but possibly due to a higher contamination rate of ICR-derived mouse embryonic fibroblast (MEF) cells used as feeder cells for coculture with GS and mGS cells. Alternatively, a mutator gene(s) defect might have increased the mutation rate, although apparent mutations were not observed in relevant DNA repair genes. The estimated mutation rate of mGS cells (excluding subclone 2 in exp1) was 0.76 to 1.42 (mean = 1.0, 95% confidence interval = 0.72–1.4) × 10^−9^ per base per cell generation (Fig. 2a). In contrast, the estimated rates of INDELs were 0.011 to 0.039 (mean = 0.024, 95% confidence interval = 0.013–0.036) × 10^−9^ and 0.048 to 0.17 (mean = 0.10, 95% confidence interval = 0.036–0.16) × 10^−9^ per base per cell generation for GS and mGS cells, respectively (Fig. 2b). These results showed that GS cells have a significantly lower mutation rate than mGS cells (*p* < 0.05, Mann–Whitney test).

### Chromosome localization of de novo mutations in GS and mGS cells

The de novo SNVs detected in GS and mGS cells were distributed across the genome at the chromosomal level (Fig. 2c). No significant enrichment was detected on a specific chromosome by analyzing the normalized counts of SNVs (SNV counts divided by the length of each chromosome × 10^8^ bases) in both GS and mGS cells (Fig. 2d). Chromosomes X and Y were excluded from this analysis, as these sex chromosomes are relatively rich in repeated sequences and exhibited highly variable sequence coverages after filtering out multimapping reads in the analytical pipeline. Similarly, de novo INDELs in both GS and mGS cells were distributed throughout the genome at the chromosomal level (Fig. 2e). The length distribution of INDELs was similar in GS and mGS cells (Fig. 2f). One-base INDELs were most frequent, whereas longer INDELs were less observed. In both GS and mGS cells, deletions were more frequent than insertions (the count ratios of insertions to deletions were approximately 0.84 for both GS and mGS cells).

**Figure 3:**
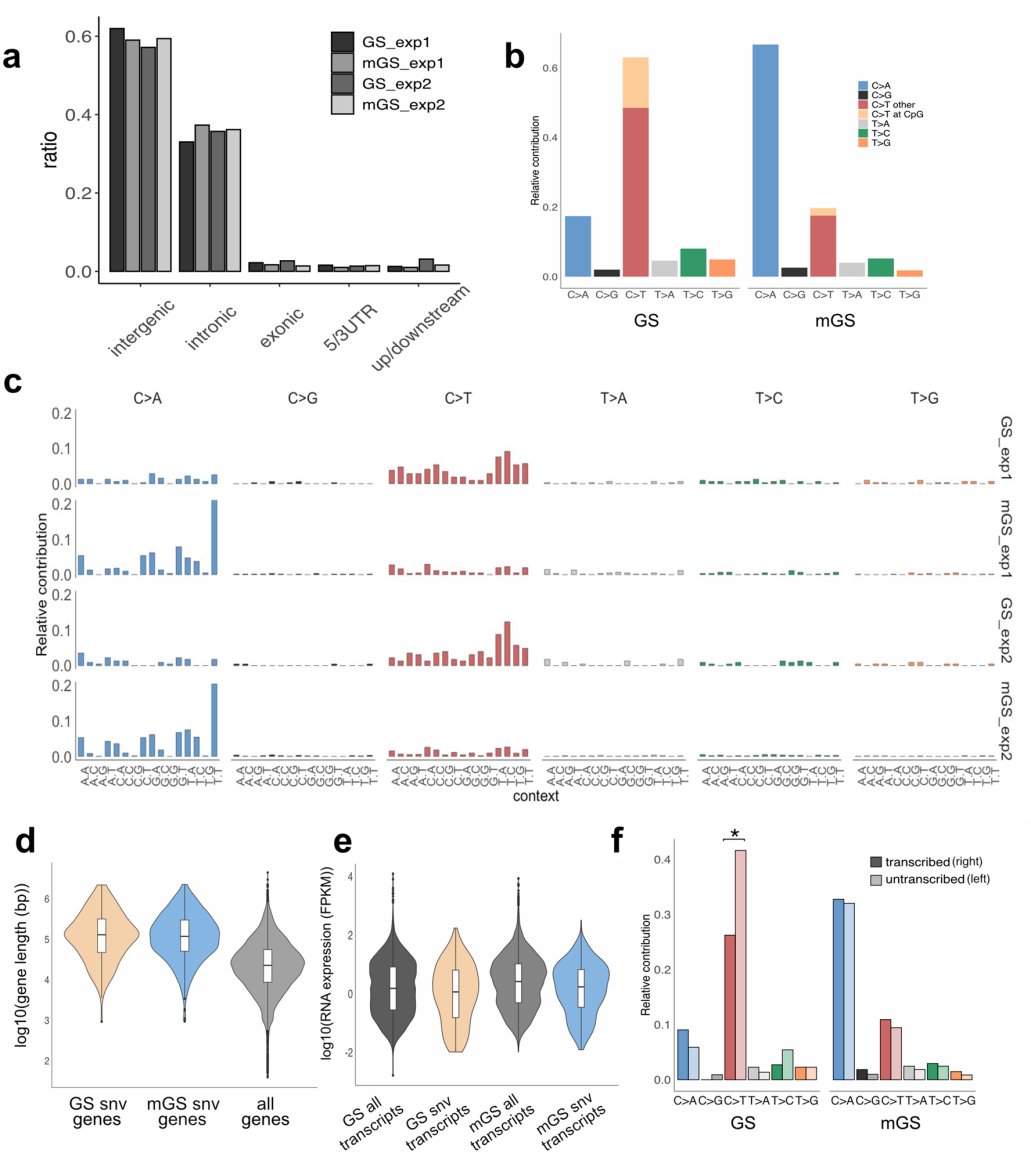
Mutational signature and correlation analysis between de novo SNVs and genetic features. (**a**) Localization of de novo SNVs in intergenic, intronic, exonic, 5′/3′-UTRs, and upstream/downstream (1 kb from transcription start/end sites) regions across the genome. Relative ratios of de novo SNV numbers in each genetic feature are shown for GS and mGS cells from exp1 and exp2. (**b**) Mutation spectrum of de novo SNVs showing the relative contribution of six mutation types (C-to-A, C-to-G, C-to-T, T-to-A, T-to-C, and T-to-G). Data of each subclone from exp1 and exp2 were combined for GS cells (left) and mGS cells (right), respectively. For C-to-T transition, CpG and other sites are separately displayed. (**c**) Mutational signatures of de novo SNVs in GS and mGS cells from exp1 and exp2. The relative contribution of 96 sequence contexts surrounding de novo SNV sites are shown for each sample. (**d**) Violin plots with box plots showing gene length (log_10_ base pairs) of genes containing de novo SNVs in GS cells (brown), mGS cells (blue), and all known genes across the genome (gray). (**e**) Violin plots with box plots showing gene expression levels (log_10_ FPKM) of genes containing de novo SNVs in GS cells (brown), mGS cells (blue), and all expressed genes in GS cells (dark gray) and mGS cells (light gray). (**f**) Transcription strand bias of mutation spectrum of de novo SNVs in GS cells and mGS cells. The relative contribution of six mutation types with a distinction of untranscribed strands (light color bars on the right of each pair of bars) and transcribed strands (dark color bars on the left of each pair of bars) is presented. The C to T substitution was more frequently observed on the untranscribed strands than the transcribed strands of the genes across the genome in GS cells (*p* < 0.05, indicated with *).

The localization of de novo SNVs was then examined within the genetic features at the gene level (Fig. 3a). De novo SNVs were most frequently observed in intergenic regions (57–62%), followed by intronic regions (33–37%) in both GS and mGS cells. The fractions of de novo SNVs in exonic (1.4–2.7%), 5′/3′-untranslated regions (UTRs; 1.0–1.6%), and upstream/downstream (1 kb from the transcription start/end sites; 1.0–3.1%) regions were relatively low. These distributions roughly correlated with the ratios of each genetic feature across the mouse genome, taking into account that the effective genome regions used in this study were relatively depleted of repetitive sequences abundant in intergenic regions. The localization patterns of de novo INDELs within the genetic features at the gene level were similar to those of de novo SNVs in both GS and mGS cells, although a larger variability was observed possibly due to the lower counts of de novo INDELs than SNVs.

Although no apparent differences were found in the localization patterns of de novo SNVs in GS and mGS cells, there was a clear difference in base substitution patterns. Among six subtypes of base substitutions [i.e., C-to-A, C-to-G, C-to-T, T-to-A, T-to-C, and T-to-G (C-to-A and G-to-T substitutions etc. are considered equivalent)], C-to-T substitution was most predominant in GS cells (63%), followed by C-to-A substitution (17%). In contrast, in mGS cells, C-to-A substitution was more predominant (66%) than C-to-T substitution (20%). Other classes of base substitutions were relatively rare in both cell types (Fig. 3b). The base substitution pattern observed in GS cells was similar to those reported for several adult somatic stem cells^18^. In contrast, the base substitution patterns of in vivo germ cells estimated from human and mouse pedigree analyses^3,4^ were also enriched in C-to-T transition, but the proportion of T-to-C transition was higher than that of GS cells (less than 10% in GS cells in vitro vs. approximately 15–25% in germ cells in vivo^4^). Unlike GS cells, the base substitution pattern of mGS cells with C-to-A transversion as the most frequent was distinct from other cell types and was characteristically reported in previous studies of human pluripotent stem cells^19–21^. Mutational signatures^22^ were further examined, which enabled a more detailed characterization of mutational processes by considering 5′ and 3′ bases surrounding SNVs (4 × 6 × 4 = 96 patterns) in GS and mGS cells (Fig. 3c). In GS cells, the most prominent SNVs were observed at the TCN (N = A, C, G, T) sequences in both exp1 and exp2. This pattern bore a resemblance to a certain degree to mutational signatures SBS7a and SBS30 in the COSMIC database (https://cancer.sanger.ac.uk/signatures/). The former was supposed to be associated with ultraviolet photoproducts and the latter with a deficiency in base excision repair (BER). In mGS cells, the most prominent SNV was observed at the TCT sequence, followed by GCT and others. These patterns were similar to mutational signatures SBS18 and SBS36 in the COSMIC database, both supposed to be associated with reactive oxygen species (ROS).

Whether de novo SNVs in GS and mGS cells might have differential characteristics with regard to transcription units was then examined. With respect to transcript length, genes that contained de novo SNVs (i.e., within exonic, intronic, 5′/3′-UTRs, and upstream/downstream regions) in both GS and mGS cells were significantly longer than all genes (regardless of the presence or absence of SNVs) across the mouse genome (Fig. 3d). This result could simply be due to the increased probability of mutation occurrence in longer genomic regions than shorter ones. Alternatively, longer transcripts may be more subject to transcription-associated mutational processes. In light of transcript abundance (Fig. 3e), the expression levels (FPKM values of RNA-seq data^23^) of genes containing de novo SNVs in GS cells were slightly lower than all genes whose expression was detected in GS cells, although the effect size [absolute mean difference divided by the standard deviation (SD)] was rather small. The same held true for mGS cells (RNA-seq data of ES cells were used as a substitute for mGS cells^23^). These observations may imply that genes with higher expression levels tend to have fewer SNVs, possibly due to transcription-coupled repair. However, the effect sizes, if any, were rather small in both GS and mGS cells. In contrast to the transcript abundance, a more distinct characteristic of transcription strand bias of de novo SNVs was observed in GS cells (Fig. 3f). When the six subtypes of base substitutions that were found in the genes across the genome were examined for whether each substitution was on the transcribed strand (the transcribed strand is the noncoding strand of each gene, which serves as a DNA template for RNA polymerase) or untranscribed strand (the untranscribed strand is the coding strand of each gene, which is not transcribed to RNA), C-to-T substitution in GS cells exhibited a clear strand bias that a significantly higher number of C-to-T substitutions were found on untranscribed strand than transcribed strand (*p* < 0.05, Poisson test). Such transcription strand bias was not significant for other substitutions in GS and mGS cells. These results suggested that the transcription bias of C-to-T substitution is a characteristic feature of GS cells compared to mGS cells.

### Copy number variations in GS and mGS cells

**Figure 4:**
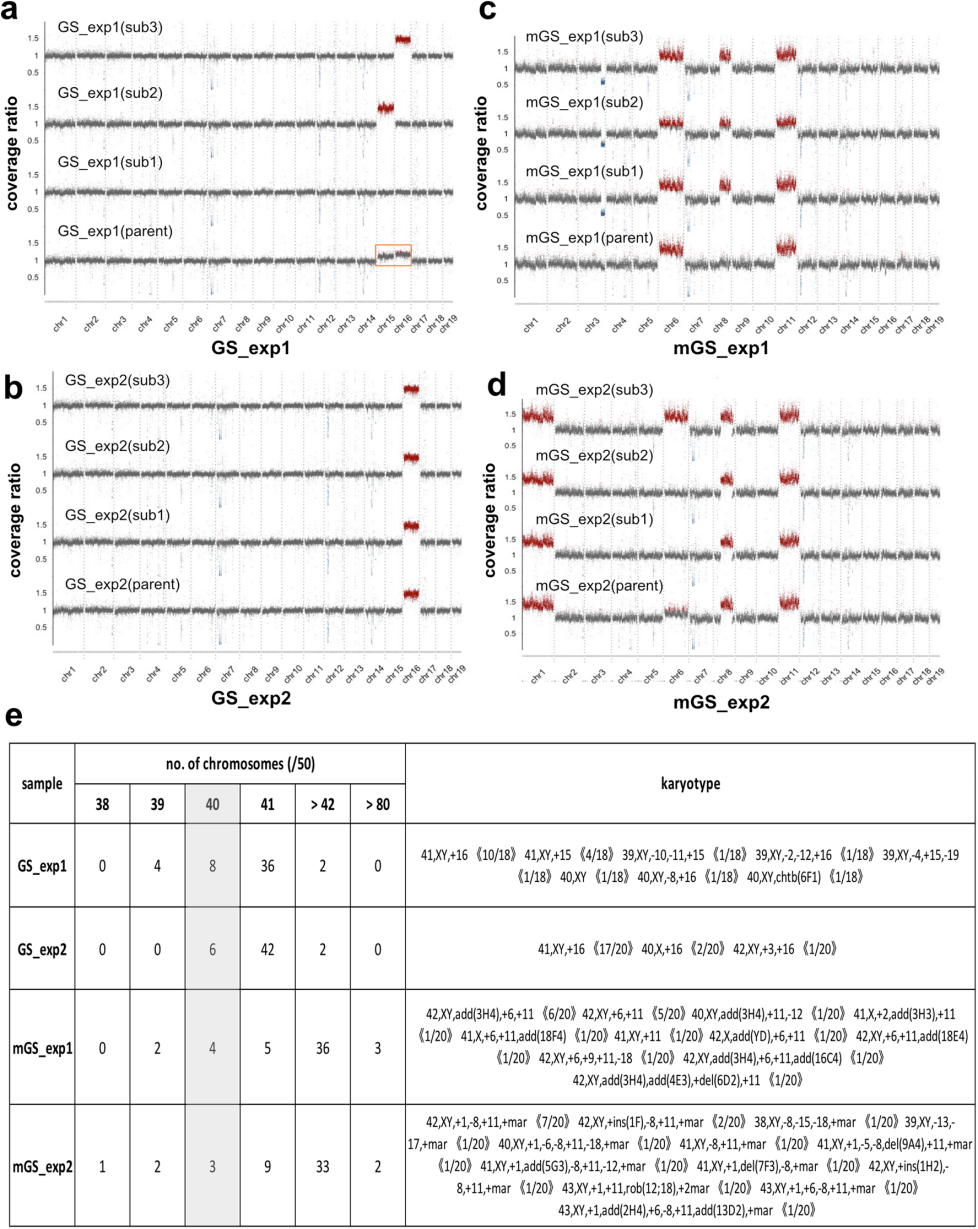
Copy number analyses of WGS data and karyotype analyses by Q-banding. (**a**–**d**) Copy number plots of WGS data across the autosomes of parental (parent) and subclone (sub1, sub2, and sub3) cultures of GS cells from exp1 (**a**), GS cells from exp2 (**b**), mGS cells from exp1 (**c**), and mGS cells from exp2 (**d**). Vertical axes represent the coverage ratios of sequence reads, with diploid regions of about 1 (gray), trisomic regions of about 1.5 (red), and monosomic regions of about 0.5 (blue). Chromosomes 15 and 16 in GS cells of the parental culture of exp1 (orange box) showed intermediate coverage ratios between 1 and 1.5, suggesting that this cell population consisted of a mixture of diploid and trisomic cells for chromosomes 15 and 16. (**e**) Summary of karyotype analyses of parental clonal cultures of GS and mGS cells from exp1 and exp2. The number of chromosomes was counted in 50 cells for each sample (2n = 40 chromosomes in mice). For karyotype analyses by Q-banding, the number of metaphase spreads showing each karyotype as indicated and the total number of metaphase spreads examined are shown in brackets. chtb, chromatid break; add, additional; del, deletion; mar, marker chromosome; ins, insertion; rob, Robertsonian translocation.

Copy number analyses were next conducted at the chromosomal level using the sequence read coverage data of WGS of parental and subclonal cultures of GS and mGS cells. In GS cells, the parental culture of exp1 [GS_exp1(parent)] exhibited mostly normal copy numbers, except for chromosomes 15 and 16, which showed slight increases in copy numbers (Fig. 4a, orange box), suggesting that this culture contained a subpopulation with the amplification of chromosomes 15 and 16. In accordance with this, among subclonal cultures of exp1, subclones 2 and 3 [GS_exp1(sub2) and GS_exp1(sub3)] exhibited trisomic amplification of chromosomes 15 and 16, respectively. In exp2 of GS cells (Fig. 4b), all parental and subclonal cultures exhibited trisomic amplification of chromosome 16.

In contrast to GS cells, copy number aberrations in mGS cells were more severe (Fig. 4c, d). All parental and subclonal cultures of mGS cells in both exp1 and exp2 exhibited trisomic amplification of chromosome 11, and seven of eight cultures had a partial amplification of chromosome 8, five of eight cultures showed amplification of chromosome 6, and four of eight cultures contained chromosome 1 amplification. Further, the three subclones in exp1 exhibited partial monosomic deletion in chromosome 3.

Based on these observations, karyotype analyses of parental cultures of GS and mGS cells were carried out in exp1 and exp2 (Fig. 4e). In GS cells, euploid cells comprised 16% and 12% of the cells examined, and the modal number of chromosomes was 41 for both parental cultures (GS_exp1 and GS_exp2 in Fig. 4e). Q-banding analysis revealed that GS_exp1 exhibited recurrent amplification of chromosomes 15 and 16, whereas GS_exp2 had chromosome 16 trisomy. These results were consistent with the copy number variations detected by WGS data (Fig. 4a, b). In contrast, karyotype analyses of mGS cells showed euploidy of 8% and 6% with the modal number of chromosomes 42. By Q-banding analyses, chromosomal aberrations were rather frequent in mGS cells, including the copy number variations detected in Fig. 4c, d. Compared to GS cells, mGS cells exhibited a much larger variety of structural abnormalities in addition to chromosomal amplifications (Fig. 4e). These results confirmed the copy number variations in GS and mGS cells detected by WGS analyses and unveiled that GS and mGS cells accumulated distinct patterns of chromosomal aberrations during clonal expansion from single cells.

### Analysis of ROS levels in low and high density cultures

Our previous study has suggested that GS cells have a relatively stable karyotype^9^. In accord with this, GS cells at early passages (approximately five to six passages after the intial establishment from neonatal testes) did not show consistent chromosomal aberrations such like trisomy of chromosomes 15 and 16, except for sporadic non clonal chromosomal aberrations (supplementary Figure 1a). One possible explanation for the recurrent chromosomal aberrations observed in clonal cultures of GS cells was increased cellular stress imposed by low cell density. A previous study has shown that low cell density cultures induced high ROS in neuronal precursor cells^24^. We thus measured ROS levels of GS cells and mGS cells under low vs high cell density cultures. However, overall ROS levels of both GS and mGS cells were not significantly higher in low cell density samples as compared to high cell density samples (mean fluorescence intensity; MFI, n = 3), while we noticed that the signal distribution of ROS levels was broader in low density cell cultures (supplementary Figure 1b). This result suggested that some of the cultured cells were exposed to higher ROS levels by plating under low density culture conditions, which might have contributed to karyotype abnormalities.

### Mutation accumulation during bulk culture of GS cells

The above results of clonogenic experiments showed that GS cells exhibited lower mutation rates and fewer chromosomal abnormalities than mGS cells, although both GS and mGS cells are germline competent stem cell resources. To further characterize the mutations that arise under a standard culture condition of GS cells, WGS analyses of GS cells maintained by bulk culture for 5, 36, and 60 months were carried out. In contrast to an artificial clonal culture experiment that allows for the estimation of mutation rates but is not usually employed as a routine culture method, de novo mutations detected during bulk culture reflect the genetic changes of the cell population as a whole, possibly arising from (1) a group of cells that acquired a certain genetic change(s) gained a growth advantage over other cells and dominated the culture or (2) a stochastic drift of growth-neutral mutations that are bottlenecked during the culture processes of a limited number of cells. Note that de novo mutations detected in bulk culture, classified as heterozygous or homozygous mutations (mutation frequencies of about 0.5 or 1.0), do not directly allow for the estimation of “mutation rates” but only reflect “mutation accumulation” as a cell population.

**Figure 5:**
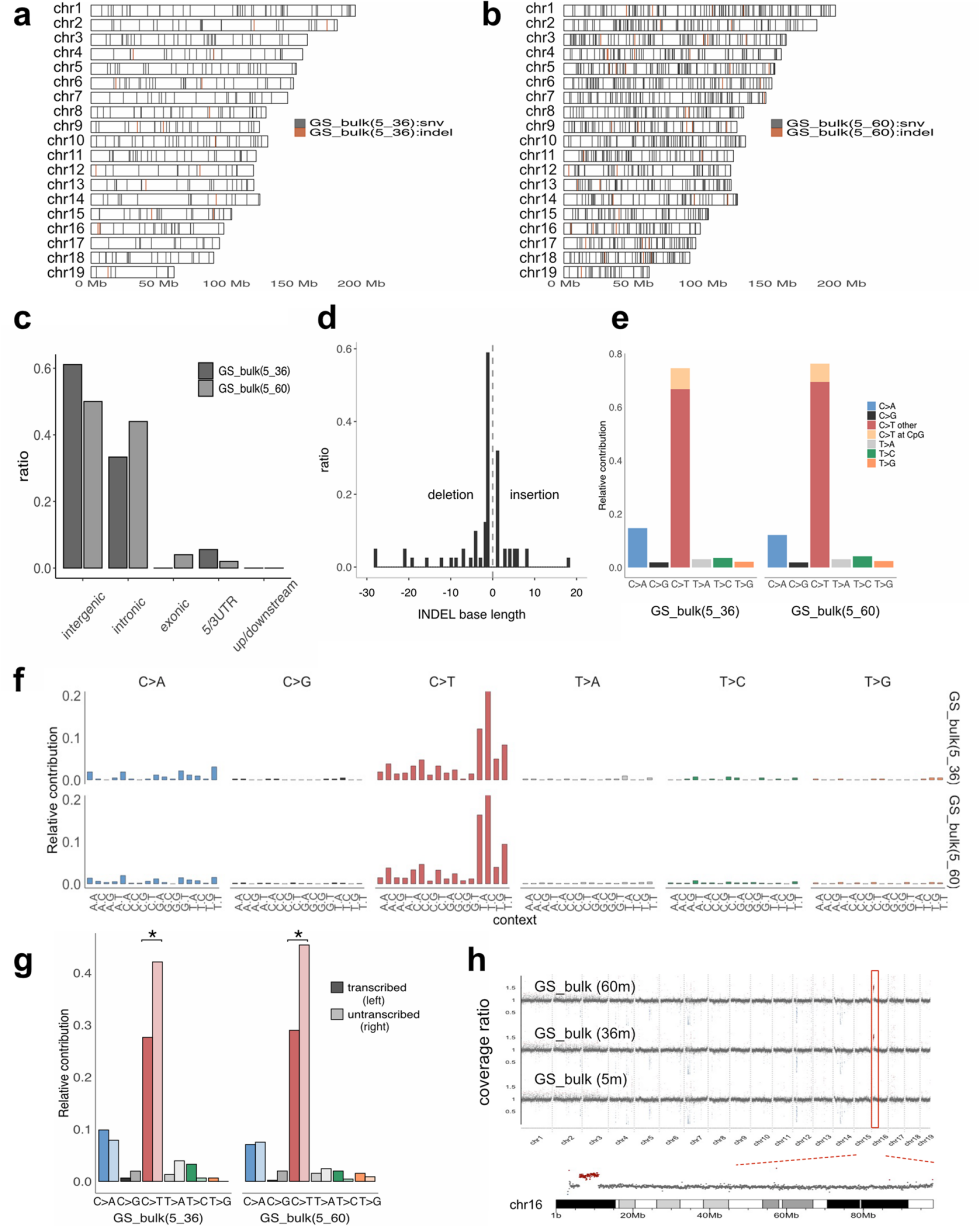
Mutation accumulation analyses during prolonged bulk culture of GS cells. (**a**, **b**) Chromosomal distribution of de novo SNVs (gray) and INDELs (red) that accumulated in GS cells during prolonged culture periods between 5 and 36 months [GS_bulk (5_36)] (**a**) and between 5 and 60 months [GS_bulk (5_60)] (**b**) after the establishment of the GS cell line. (**c**) Localization of de novo SNVs in intergenic, intronic, exonic, 5'/3'-UTRs, and upstream/downstream (1 kb from transcription start/end sites) regions across the genome in prolonged bulk culture of GS cells. The samples are the same as in (**a**) and (**b**). (**d**) Base-pair length of de novo INDELs [insertions on the right (> 0) and deletions on the left (< 0)] detected in prolonged bulk culture of GS cells. Data from GS_bulk (5_36) and GS_bulk (5_60) were combined due to the low counts of de novo INDELs in this experiment. (**e**) Mutation spectrum of de novo SNVs detected in GS_bulk (5_36) and GS_bulk (5_60) showing the relative contribution of six mutation types (C-to-A, C-to-G, C-to-T, T-to-A, T-to-C, and T-to-G). For C-to-T transition, CpG and other sites are separately displayed. (**f**) Mutational signatures of de novo SNVs detected in GS_bulk (5_36) and GS_bulk (5_60). The relative contribution of 96 sequence contexts surrounding de novo SNV sites is shown for each sample. (**g**) Transcription strand bias of mutation spectrum of de novo SNVs detected in GS_bulk (5_36) and GS_bulk (5_60). The relative contribution of six mutation types with a distinction of untranscribed strands (light color bars on the right of each pair of bars) and transcribed strands (dark color bars on the left of each pair of bars) is shown. The C to T substitution was more frequently observed on the untranscribed strands than the transcribed strands of the genes across the genome in GS cells (*p* < 0.05, indicated with *). (**h**) Copy number plots of WGS data across the autosomes of GS cells after prolonged bulk culture for 5, 36, and 60 months (top). Chromosome 16 showed a segmental amplification spanning between 6.05 and 11.1 MB after prolonged culture of 36 and 60 months (bottom). Genes present in this amplified region (plotted in red) are summarized in Supplementary Table 11.

GS cells at 5 months of bulk culture after initial establishment were used as a stable working stock of the cell line, and de novo mutations that accumulated after 36 and 60 months of bulk culture compared to 5 months of culture were analyzed by WGS using the same bioinformatics pipeline as described above. The estimated number of de novo SNVs detected between 5 and 36 months and between 5 and 60 months [designated as GS_bulk (5_36) and GS_bulk (5_60) hereafter] was 427 and 1195 (332 overlaps), and the estimated number of de novo INDELs of GS_bulk (5_36) and GS_bulk (5_60) was 19 and 51 (9 overlaps), respectively. The chromosomal distribution of these SNVs and INDELs is shown in Fig. 5a and b.

The localization patterns of de novo SNVs in bulk culture in genic features, such as exons, introns, intergenic regions (Fig. 5c), were similar to those observed for clonal cultures of GS cells (Fig. 3a), although some variations were observed. With regard to exonic mutations, as aged GS cells proliferate faster than young GS cells^11^, GS cells after prolonged bulk culture may likely carry mutated genes that might contribute to enhanced proliferation. Mutations in *Fgfr*, *Ret*, and *Shp2* involved in the self-renewal of GS cells or paternal effect^25,26^ were analyzed, but none of these genes showed de novo mutations. Also, because *Trp53* mutations are often found in human pluripotent stem cell cultures^17^, mutations relevant to *Trp53* were checked. However, no mutations were found in *Trp53* or related pathways after bulk culture of GS cells similarly to clonally expanded (parental and subuclonal) GS cell lines.

At the base level, the substitution patterns of SNVs (Fig. 5e) and the length distribution of INDELs (Fig. 5d) were mostly similar to those observed in clonal cultures of GS cells (Figs. 2f and 3b), except that the proportion of C-to-T substitution was more prominent than other types of substitutions in bulk culture (Fig. 5e). This may imply a shift in the mutation pattern or DNA repair activity or reflect technical differences in culture conditions between clonal and bulk cultures, although no experimental evidence is currently available to explain this observation.

The mutational signatures of de novo SNVs that accumulated during bulk culture of GS cells (Fig. 5f) were close to those observed in clonal cultures (Fig. 3c), but the fractions of TCN (N = A, T, G, C) were more increased in accordance with the elevated substitution rate of C-to-T, as described above (Fig. 5e). Concerning the transcription strand bias for C-to-T transition, statistical significance was again observed (*p* < 0.05, Poisson test) in bulk cultures of GS cells (Fig. 5g), similar to clonal cultures (Fig. 3f). These results indicated that the observed features (i.e., the mutational signature and the transcription strand bias for C-to-T transition) are characteristic of GS cells regardless of culture conditions.

Finally, copy number alterations in GS cells after prolonged bulk culture were examined using the genome coverage data of WGS (Fig. 5h). At 5 months of culture, no copy number aberrations at the chromosomal level were detected. However, after 36 and 60 months of culture, a segmental amplification was observed for the 6.05 to 11.1 MB region of chromosome 16. The list of known transcripts localized on this amplified region of chromosome 16 is summarized in Supplementary Table 11. Together with the recurrent trisomy of chromosome 16 in clonal cultures of GS cells (Fig. 4a, b, e), these data suggested that chromosome 16, and most likely the amplified region between 6.05 and 11.1 MB, drives GS cell proliferation in culture.

## Discussion

The germline lineage is unique because it transmits genetic information to the next generation. Despite its importance, little data are available to direct measurement of the types and rates of germline mutations. In classic experiments, these were evaluated by phenotypes of the offspring. For example, the frequency of mutations in specific seven recessive markers was evaluated after irradiation to study the impact of radiation-induced DNA damage in mice^27^. However, such analyses require a large number of animals and preclude germ cell analysis before fertilization. The development of the GS cell culture technique allowed us to collect a large number of germ cells for DNA analyses. In addition, WGS greatly facilitates quantitative analysis of genome mutations with high fidelity. These two techniques were used to study the impact of cell culture on the genome mutations in germline cells.

Several previous studies have suggested a lower mutation rate in the germline compared to somatic cells. For example, by seminal population genetic studies^1^, Lynch showed that the mutation rate of human germline is rather low (0.06 × 10^−9^/base/cell division) compared to various somatic lineages (0.27 × 10^−9^–1.47 × 10^−9^/base/cell division). Recent WGS analyses of pedigrees of humans and mice^2–4^ then provided detailed theoretical estimates of germline mutation rates (0.033 × 10^−9^ and 0.056 × 10^−9^ for humans and 0.12 × 10^−9^ and 0.09 × 10^−9^ for mice, from the studies by Milholland et al. (2017)^3^ and Lindsay et al. (2019)^4^ respectively), which were again significantly lower than somatic cells^3^ and also early embryonic cells^4^. Our study now reports a direct measurement of mutation rates of mouse SSCs using clonogenic cultures of GS cells. The value was rather low (approximately 0.22 × 10^−9^/base/cell division) but still 2.4- to 3.9-fold higher than the values estimated for mouse germ cells by pedigree analyses, as described above^3,4^. This discrepancy may stem from differences in experimental conditions. One possibility is that in vivo germ cells and the offspring derived thereof are selected based on fitness during gametogenesis and development, whereas GS cells in culture are not subject to such selection. Another possibility is that mutational stress, such as high oxygen comsumption or ROS, elevated temperature etc., is more highly imposed on germ cells in culture than those in vivo. In relation to this, chick primordial germ cells that were expanded in culture were recently analyzed by WGS^28^, with the mutation rate of 0.27 × 10^−9^/base/cell division, which was close to the value of mouse GS cells in this study. Future development to track clonal cell expansion and divisions of germ cells in vivo would directly compare mutation rates in vitro and in vivo.

Similar to germ cells, the mutation rates of pluripotent stem cells were lower than those of somatic cells^19–21,29^, although it was unclear whether pluripotent stem cells were genetically as stable as germ cells. This analysis now showed a higher mutation rate in mGS cells than GS cells. mGS cells were derived spontaneously during the propagation of GS cells. Although the frequency of spontaneous generation of mGS cells is low (approximately 1 of 30 testes), the simultaneous transfection of GS cells with short hairpin RNA against *Dmrt1* and *Trp53*^30^, both known suppressors of teratomas, significantly enhances the derivation efficiency. Using mGS cells, they could contribute to the germline by microinjection into blastocysts after gene targeting. In contrast, they formed teratoma when microinjected into seminiferous tubules. Therefore, mGS cells no longer maintain the ability to recolonize seminiferous tubules to reinitiate spermatogenesis but are converted to pluripotent stem cells with germline competence. The only known difference between mGS and ES cells is the DNA methylation pattern^10^. Whereas ES cells have a somatic cell DNA methylation pattern in imprinted genes, mGS cells have an androgenetic imprinting pattern. Because ES and mGS cells are very similar in their phenotype, the results raised a possibility that pluripotent stem cells actually have elevated mutation rates than SSCs, although mutation data of WGS for mouse ES cells are currently unavailable. Note that differences in genetic background likely influence the mutation rates of inbred mouse strains. For example, DNA polymerase ι (polι) is mutated in 129 mice^31^. Such differences may influence the analysis of mutation rates. Further, pluripotent stem cells are cultured under several distinct conditions that reflect different developmental stages, such as naïve, intermediate (metastable), and primed states^32–34^. Mouse mGS cells used in this study were cultured under the classic but still widely used intermediate state condition, whereas human pluripotent stem cells are most commonly maintained under the primed state condition. Further studies are necessary to clarify whether such differences in developmental states of pluripotent stem cells affect mutation rates and patterns.

The mechanism by which GS cells achieve a low mutation rate is currently unknown. It was recently found that GS cells exhibit a high BER activity^35^. Unlike other stem cells, GS cells are relatively resistant to H_2_O_2_, which induces not only double-strand breaks but also point mutations. ROS are required for SSC self-renewal because GS cells derived from mice deficient in *Nox1*, which generates ROS, have a significantly lower self-renewal rate in vivo^36^. Whereas ES cells are extremely sensitive to H_2_O_2_ and undergo apoptosis, GS cells could tolerate high doses of H_2_O_2_. Our previous analysis revealed that GS cells express *Ogg1* strongly compared to ES or MEF cells. *Ogg1* is responsible for the removal of oxidized base^37^. It recognizes the damaged base and excises it from the DNA strand. Because it is induced by H_2_O_2_ in GS cells, *Ogg1* may play a role in the protection against DNA damage during culture. Because no such high expression of *Ogg1* was found in mGS cells, this difference in *Ogg1* expression levels might have increased mutation rates in mGS cells.

In addition to mutation rates, GS and mGS cells exhibited distinct patterns of base substitutions. Base substitution patterns reflect mutagenic processes, including the types and levels of genotoxic stresses and those of DNA repair pathways^22^. Our results showed that the mutation pattern in GS cells was similar to several somatic stem cells^18^. The major cause of C-to-T transition, which was most frequent in GS cells, is the spontaneous deamination of methylated and unmethylated C. This type of transition is commonly observed in various cell types^18^. In contrast, the C:G to A:T transversion prominent in mGS cells is characteristic of pluripotent stem cells^19–21^. This transversion possibly reflects high oxidation stress in rapidly proliferating cells, which generates 8-oxo-2′-deoxyguanosine, resulting in G-to-T transversion after DNA replication^38^.

With regard to C-to-T transition in GS cells, a transcription strand bias was found for this type of base substitution. It is proposed that the untranscribed strand is more subject to transcription-associated DNA damages, such as deamination of C-to-T, as a result of single-strand exposure during transcription, whereas the transcribed strand associated with RNA polymerase complexes is processed by transcription-coupled repair when RNA transcription is impaired at damaged sites^39^. It is currently unknown why C-to-T transition, but not other base substitutions, exhibited a transcription strand bias in GS cells and not in mGS cells. It was supposed that GS and mGS cells have several distinct features of mutational stresses and/or DNA repair activities, which are worth experimental validation in future investigations.

Distinct karyotype abnormalities were found in both GS and mGS cells. Karyotype abnormalities in mGS cells resembled those in ES cells, including trisomies 8 and 11. Trisomy 11 was also found in a previous study of mGS cells after double knockout of the *Ocln* gene^40^. In contrast, GS cells showed trisomies 15 and 16. This was unexpected because previous studies have shown that GS cells maintained a relatively stable chromosome number. The only exception was when a partial deletion of chromosome 17 was noted after 5 years of culture^11^. Although it is currently unknown why GS cells in this study exhibited frequent karyotype abnormalities, it was speculated that low-density cultures might have caused this problem. It was reported previously that low cell density cultures induced high levels of ROS in neural precursor cells^24^, but GS cells as well as mGS cells under low cell density cultures did not show such overall increase in ROS levels. Instead, ROS levels were more heterogeneous in low cell density, and such heterogeneity may expose a small population of cells to high or low ROS levels, which can influence cellular proliferation rate and/or chromosomal stability. In the future, it will be of interest to test in more detail whether low cell density cultures consistently cause karyotype abnormalities in GS cells and if so to address whether trisomy of chromosomes 15 and 16 has selective growth advantage under low cell density culture. Karyotype abnormalities may also underlie the loss of differentiation (i.e., spermatogenesis) capacity of GS cells after long term culture, as observed in a previous study^11^.

In this study, WGS was used to test the hypothesis that germ cells have a lower mutation rate than somatic cells. The results confirmed previous studies showing low mutation rate estimates in spermatogonia^1,3,4,42^ and also suggested that pluripotent stem cells have an elevated mutation rate compared to spermatogonia. Although human GS cells are currently not available, it is expected that they may be useful for restoring fertility in boys whose fertility is impaired by cancer treatment. In this sense, understanding the degree and type of DNA damage in SSCs is important for the future application of SSCs for male infertility treatment^43^. Studies in ES cells have shown that significant improvement in mutation rate can occur by modifying the culture conditions^19^. Therefore, future studies to assess the impact of long-term culture and develop methods to minimize mutations are required at this stage of research. Such studies will provide a rational strategy to assess the potential risk and possibilities of safe clinical implementation of SSC-based therapies.

## Materials and methods

All methods were carried out in accordance with relevant guidelines and regulations.

### Cell culture

For clonal culture analysis, GS cells were derived from wild-type DBA/2 mice using procedures described previously^8^. mGS cells derived spontaneously during GS cell culture^10^ were also used. Both GS and mGS cells were established in the T. S. laboratory. In brief, neonatal DBA/2 mice were sacrificed by carbon dioxygen and their testes were dissociated into single cells by a two-step enzymatic digestion procedure using collagenase type IV and trypsin (Sigma). Cells were plated on 0.1% gelatin-coated culture dish and incubated overnight. On the next day, cells were transferred on MEF cells derived from 13.5 days postcoitum ICR embryos and expanded using Stempro 34 medium (Thermo Fisher Scientific) supplemented with rat GDNF and human FGF2 (Peprotech). Iscove's modified Dulbecco's medium and knockout serum replacement (Thermo Fisher Scientific) supplemented with rat GDNF and human FGF2 was used for clonal culture. mGS cells were derived spontaneously during GS cell derivation. After conversion into ES cell-like colonies, mGS cells were maintained on MEF cells in standard ES cell culture medium containing 10% fetal bovine serum and leukemia inhibitory factor (ESGRO, Merck Millipore). For single-cell cloning, cells were initially plated in 6-well plates, and then individual colonies were transferred to a 96-well plate. These cells were allowed to replicate for 100 cell population doublings. The number of cells was recorded at each passage. For genome DNA sampling, GS and mGS cultures were transferred to culture dishes coated with 1% gelatin and then incubated for 30 min to remove MEF cells. After repeating this procedure three times, the proportion of contaminating MEF cells was less than 1%. For bulk cultures, GS cells derived from C57BL6/Tg14(act-EGFP-OsbY01) on a DBA/2 background were used^8^. All animal experiments were conducted according to the ethical guidelines approved by the Animal Care and Use Committee of Kyoto University Graduate School of Medicine with consideration of both the scientific rationale and the welfare of the animal. This study was performed in accordance with ARRIVE guidelines (https://arriveguidelines.org).

### WGS

Genome DNA was extracted from frozen cell pellets of GS and mGS cells (~ 10^6^ cells/sample) in a lysis buffer containing 15 mM Tris-HCl, 10 mM EDTA, 0.75% sodium dodecyl sulfate, and 0.25 mg/ml proteinase K at 55 °C for overnight, followed by phenol/chloroform extraction and ethanol precipitation. Genome DNA quantified by PicoGreen (Thermo Fisher) and checked by gel electrophoresis and Bioanalyzer (Agilent) was fragmented by the Covaris focused ultrasonicator. WGS libraries were prepared using the TruSeq DNA PCR Free (350) kit and TruSeq DNA LT sample prep kit (Illumina) for clonal and bulk culture samples, respectively. The raw sequence data of 150 and 100 bp paired-ends were obtained using NovaSeq and HiSeq sequencing systems (Illumina) for clonal and bulk culture samples. Base call from raw images was carried out by the Illumina real-time analysis pipeline. The read depth of each sample was approximately 25 to 30 × genome coverages.

### Bioinformatic analysis

To quality-control raw FASTQ sequences, FastQC (Babraham Bioinformatics) was used, followed by Trimmomatic^44^ to trim low-quality reads and bases. Genome mapping of trimmed FASTQ sequences was carried out using the Burrows–Wheeler Aligner^45^ to the mouse GRCm38 (mm10) reference genome sequence. After quality filtering of mapped reads by MAPQ and FLAG values of SAM files by AWK, Samtools^46^ was applied to fixmate and remove potential PCR duplicates. Strelka2^47^ was run in combination with Manta^48^ to call de novo SNVs and INDELs by comparative analyses between control and sample sequences (parental vs. subclones in clonal cultures of GS and mGS cells and 5 vs. 36 months and 5 vs. 60 months for bulk cultures of GS cells).

After quality pass filtering of SNVs and INDELs with bcftools^49^, genomic regions with less than 10 sequence reads for each sample were removed to obtain high fidelity mutation calling, and somatic EVS values of the Manta Strelka2 pipeline were used to filter low-quality calls (thresholds were 19 for SNVs and 14 for INDELs). Known single nucleotide polymorphisms and small INDELs of more than 30 mouse inbred strains (the Mouse Genome Project, the Wellcome Sanger Institute) were also excluded^50^, including those from which GS and mGS cells were established, using the outer join function of Postgresql. Such filtering of known polymorphic sites in various mouse strains reduced false-positive calls potentially arising from the strain difference between the reference genome and our cell lines and potential contamination of MEF cells derived from ICR closed colony mice, which were used as a feeder layer of GS and mGS cultures. Although MEF cells were removed by a differential attachment method to gelatin-coated culture dishes before genome extraction of GS and mGS cells, and the level of contamination was estimated to be less than 1%, such a low level of cellular heterogeneity is known to still give rise to a substantial number of false-positive mutation calls, especially at low allele frequencies^51^.

De novo mutations that accumulate in each cell division during the expansion of parental clonal culture should exhibit allele frequencies of about 0.5 and 1.0 in subclonal cohorts, whereas de novo mutations that appear during the expansion of subclonal cultures show mutation frequencies of less than 0.5 (0.25, 0.125, etc.) in subclonal cohorts. A potential heterogeneity of cell population, such as contamination of feeder cells, also gives rise to mutations at low allele frequencies. Thresholds to filter out mutations at low allele frequencies (0.3 for GS and 0.4 for mGS cells) were defined by visual inspection of the histograms of allele frequencies of each sample, and a Gaussian distribution model was then applied to estimate the total number of mutations with allele frequencies of 0 to 1. The mutation ratios were calculated by dividing these estimated numbers of mutations by twice (diploid) the length of effective genome regions that had more than 10 read coverage in both control (parental) and sample (subclones) sequences (approximately 2–2.2 × 10^9^ for a haploid) and by the number of population doublings (100). Gene annotations for SNVs were obtained using Annovar^52^. Mutational signatures were analyzed using MutationalPatterns^53^ in R. Chromosome distributions of SNVs and INDELs were visualized by ggbio and karyoplotteR^54^ in R. Copy number alterations at the chromosomal level were analyzed using Control-FREEC^55^ with the resulting data plotted by R. Chromosomes X and Y were excluded from copy number analyses because read depths mapping to chromosomes X and Y were highly variable in our analysis pipeline that strictly excluded multimapping reads. The transcript list in the amplified region of chromosome 16 was obtained using the Table Browser of the University of California-Santa Cruz. RNA-seq data of GS and ES cells were obtained from a previous report^23^.

### Karyotype analysis

For karyotype analyses, GS and mGS cells were cultured in the presence of M-phase arresting solution II containing colcemid and vinblastine (ChromosomeScienceLabo, Japan) to enrich for metaphase cells, followed by 0.25% trypsin and 0.5 mM EDTA treatment to prepare single-cell suspensions. The cell suspensions were then treated with Hypotonic Solution II (ChromosomeScienceLabo), followed by fixation with Carnoy’s solution (3:1 methanol/acetic acid). Metaphase spreads were prepared by dropping the cell suspension onto slide glasses (Muto Pure Chemicals, Japan), air-drying, and then staining with Q-banding reagents containing Hoechst 33258 and quinacrine (ChromosomeScienceLabo). Slides were inspected using the Leica Cytogenetic Workstation CW4000 with a Leica DC 350FX cooled CCD camera. Fifty cells were counted for chromosome numbers, and karyotyping was carried out for up to 20 cells for each sample.

### ROS measurement

To measure relative ROS levels, GS and mGS cells, cultured on laminin (iMatrix-511, Nippi, Japan)- and gelatin (Sigma)-coated dishes respectively, were plated at low (5 × 10^2^ cells/cm^2^) and high (5 × 10^4^ cells/cm^2^) cell densities. On the next day, cells were dissociated with 0.25% trypsin supplemented with EDTA in phosphate-buffered saline (PBS), washed with fetal bovine serum (FBS)-containing culture media, and resuspended in 0.1% FBS in PBS at a density of 1 × 10^4^ cells/300 μL for both low and high density culture samples. 1 µM of CM-H2DCFDA (Thermo Fisher Scientific) were added to the cell suspensions, incubated in the dark for 20 min at 37 °C, washed and resuspended in 0.1% FBS in PBS, then fluorescent signals were analysed by Guava easyCyte flow cytometer and the InCyte software (Millipore) with 488 nm excitation and around 525 nm emission detection.

### Statistical analysis

The Mann–Whitney test was used under the null hypothesis that there were no differences in the numbers of SNVs and INDELs between GS and mGS cells (Fig. 2a, b), between gene length (Fig. 3d) and gene expression (Fig. 3e) of genes containing SNVs in GS or mGS cells and all genes across the genome. The Kruskal–Wallis test was used to examine a possible enrichment of SNVs on a specific chromosome (Figs. 2d and 5a, b). The transcription strand bias was tested by the Poisson test (Figs. 3f and 5g). A Gaussian distribution model was applied to estimate the total numbers of SNVs and INDELs (allele frequency = 0–1) from trimmed data in which SNVs and INDELs with low allele frequencies were excluded (the threshold was 0.3 for clonal cultures of GS cells, 0.4 for clonal cultures of mGS cells, and 0.4 for bulk cultures of GS cells). For the FACS data, the Mann–Whitney test was used for analysis of MFI and Levene's test was employed to evaluate the equality of variances (supplementary Figure 1b). All statistical analyses were carried out using R version 3.6.

## Supplementary Information

Supplementary Figure 1.

Supplementary Table 1.

Supplementary Table 2.

Supplementary Table 3.

Supplementary Table 4.

Supplementary Table 5.

Supplementary Table 6.

Supplementary Table 7.

Supplementary Table 8.

Supplementary Table 9.

Supplementary Table 10.

Supplementary Table 11.

Supplementary Legends.

## Data Availability

The SNVs and INDELs with gene annotations by ANNOVAR are summarized in Supplementary Tables 1 to 10. The transcripts present in the 6.05 to 11.1 MB region of mouse chromosome 16 are listed in Supplementary Table 11. All FASTQ files of WGS data used in this study are available at the National Center for Biotechnology Information Short Read Archive under accession number PRJNA733604.

## References

[1] Lynch M (2010). Evolution of the mutation rate. Trends Genet..

[2] Rahbari R (2016). Timing, rates and spectra of human germline mutation. Nat. Genet..

[3] Milholland B (2017). Differences between germline and somatic mutation rates in humans and mice. Nat. Commun..

[4] Lindsay SJ, Rahbari R, Kaplanis J, Keane T, Hurles ME (2019). Similarities and differences in patterns of germline mutation between mice and humans. Nat. Commun..

[5] De Rooij DG (2017). The nature and dynamics of spermatogonial stem cells. Development.

[6] Meistrich, M. & van Beek, M. Spermatogonial stem cells. in *Cell and Molecular Biology of the Testis* (eds. Desjardins, C. & Ewing, L.) 266–295 (Oxford University Press, 1993).

[7] Tagelenbosch RAJ, de Rooij DG (1993). A quantitative study of spermatogonial multiplication and stem cell renewal in the C3H/101 F1 hybrid mouse. Mutat. Res..

[8] Kanatsu-Shinohara M (2003). Long-term proliferation in culture and germline transmission of mouse male germline stem cells. Biol. Reprod..

[9] Kanatsu-Shinohara M (2005). Genetic and epigenetic properties of mouse male germline stem cells during long-term culture. Development.

[10] Kanatsu-Shinohara M (2004). Generation of pluripotent stem cells from neonatal mouse testis. Cell.

[11] Kanatsu-Shinohara M (2019). Aging of spermatogonial stem cells by Jnk-mediated glycolysis activation. Proc. Natl. Acad. Sci. USA.

[12] Longo L, Bygrave A, Grosveld FG, Pandolfi PP (1997). The chromosome make-up of mouse embryonic stem cells is predictive of somatic and germ cell chimaerism. Transgenic Res..

[13] Liu X (1997). Trisomy eight in ES cells is a common potential problem in gene targeting and interferes with germ line transmission. Dev. Dyn..

[14] Humpherys D (2001). Epigenetic instability in ES cells and cloned mice. Science.

[15] Ishii K (2014). The Trp53-Trp53inp1-Tnfrsf10b pathway regulates the radiation response of mouse spermatogonial stem cells. Stem Cell Reports.

[16] Tichy ED, Stambrook PJ (2008). DNA repair in murine embryonic stem cells and differentiated cells. Exp. Cell Res..

[17] Merkle FT (2017). Human pluripotent stem cells recurrently acquire and expand dominant negative P53 mutations. Nature.

[18] Blokzijl F (2016). Tissue-specific mutation accumulation in human adult stem cells during life. Nature.

[19] Thompson, O. *et al.* Low rates of mutation in clinical grade human pluripotent stem cells under different culture conditions. *Nat. Commun.* **11** (2020). 10.1038/s41467-020-15271-3 PMC7089967 32251294

[20] Rouhani, F. J. *et al.* Mutational History of a human cell lineage from somatic to induced pluripotent stem cells. *PLoS Genet.* **12**, e1005932 (2016). 10.1371/journal.pgen.1005932 PMC4824386 27054363

[21] Kuijk E (2020). The mutational impact of culturing human pluripotent and adult stem cells. Nat. Commun..

[22] Alexandrov LB (2013). Signatures of mutational processes in human cancer. Nature.

[23] Ishiguro, K. *et al.* MEIOSIN directs the switch from mitosis to meiosis in mammalian germ cells. *Dev. Cell* **52**, 429–445 (2020). 10.1016/j.devcel.2020.01.010 32032549

[24] Limoli CL (2004). Cell-density-dependent regulation of neural precursor cell function. Proc. Natl. Acad. Sci. USA.

[25] Crow JF (2000). The origins, patterns and implications of human spontaneous mutation. Nat. Rev. Genet..

[26] Tartaglia M (2004). Paternal germline origin and sex-ratio distortion in transmission of PTPN11 mutations in Noonan syndrome. Am. J. Hum. Genet..

[27] Russell LB, Russell WL (1996). Spontaneous mutations recovered as mosaics in the mouse specific-locus test. Proc. Natl. Acad. Sci. USA.

[28] Woodcock ME (2019). Reviving rare chicken breeds using genetically engineered sterility in surrogate host birds. Proc. Natl. Acad. Sci. USA.

[29] Cervantes RB, Stringer JR, Shao C, Tischfield JA, Stambrook PJ (2002). Embryonic stem cells and somatic cells differ in mutation frequency and type. Proc. Natl. Acad. Sci. USA.

[30] Takashima S (2013). Regulation of pluripotency in male germline stem cells by Dmrt1. Genes Dev..

[31] McDonald JP (2003). 129-Derived strains of mice are deficient in DNA polymerase ι and have normal immunoglobulin hypermutation. J. Exp. Med..

[32] Ying QL (2008). The ground state of embryonic stem cell self-renewal. Nature.

[33] Tesar PJ (2007). New cell lines from mouse epiblast share defining features with human embryonic stem cells. Nature.

[34] Brons IGM (2007). Derivation of pluripotent epiblast stem cells from mammalian embryos. Nature.

[35] Mori Y (2021). OGG1 protects mouse spermatogonial stem cells from reactive oxygen species in culture. Biol. Reprod..

[36] Morimoto H (2013). ROS are required for mouse spermatogonial stem cell self-renewal. Cell Stem Cell.

[37] Klungland A, Bjelland S (2007). Oxidative damage to purines in DNA: Role of mammalian Ogg1. DNA Repair.

[38] Markkanen E (2017). Not breathing is not an option: How to deal with oxidative DNA damage. DNA Repair.

[39] Polak P, Querfurth R, Arndt PF (2010). The evolution of transcription-associated biases of mutations across vertebrates. BMC Evol. Biol..

[40] Takehashi M (2007). Production of knockout mice by gene targeting in multipotent germline stem cells. Dev. Biol..

[41] Wang CY, Liu LN, Zhao ZB (2013). The role of ROS toxicity in spontaneous aneuploidy in cultured cells. Tissue Cell.

[42] Murphey, P., McLean, D. J., McMahan, C. A., Walter, C. A. & McCarrey, J. R. Enhanced genetic integrity in mouse germ cells. *Biol. Reprod.* **88** (2013). 10.1095/biolreprod.112.103481 PMC4434944 23153565

[43] Kubota H, Brinster RL (2018). Spermatogonial stem cells. Biol. Reprod..

[44] Bolger AM, Lohse M, Usadel B (2014). Trimmomatic: A flexible trimmer for Illumina sequence data. Bioinformatics.

[45] Li H, Durbin R (2009). Fast and accurate short read alignment with Burrows-Wheeler transform. Bioinformatics.

[46] Li H (2009). The sequence alignment/map format and SAMtools. Bioinformatics.

[47] Kim S (2018). Strelka2: Fast and accurate calling of germline and somatic variants. Nat. Methods.

[48] Chen X (2016). Manta: Rapid detection of structural variants and indels for germline and cancer sequencing applications. Bioinformatics.

[49] Danecek P (2021). Twelve years of SAMtools and BCFtools. Gigascience.

[50] Keane TM (2011). Mouse genomic variation and its effect on phenotypes and gene regulation. Nature.

[51] Cibulskis K (2011). ContEst: Estimating cross-contamination of human samples in next-generation sequencing data. Bioinformatics.

[52] Wang, K., Li, M. & Hakonarson, H. ANNOVAR: Functional annotation of genetic variants from high-throughput sequencing data. *Nucl. Acids Res.* **38**, e164 (2010). 10.1093/nar/gkq603 PMC2938201 20601685

[53] Blokzijl F, Janssen R, van Boxtel R, Cuppen E (2018). MutationalPatterns: Comprehensive genome-wide analysis of mutational processes. Genome Med..

[54] Gel B, Serra E (2017). KaryoploteR: An R/bioconductor package to plot customizable genomes displaying arbitrary data. Bioinformatics.

[55] Boeva V (2012). Control-FREEC: A tool for assessing copy number and allelic content using next-generation sequencing data. Bioinformatics.

