# Supplementary figures and images for: Genomic stability of mouse spermatogonial stem cells in vitro
Source: Sci Rep. 2021 Dec 17;11:24199. doi: 10.1038/s41598-021-03658-1 (PMC8683475; doi:10.1038/s41598-021-03658-1)

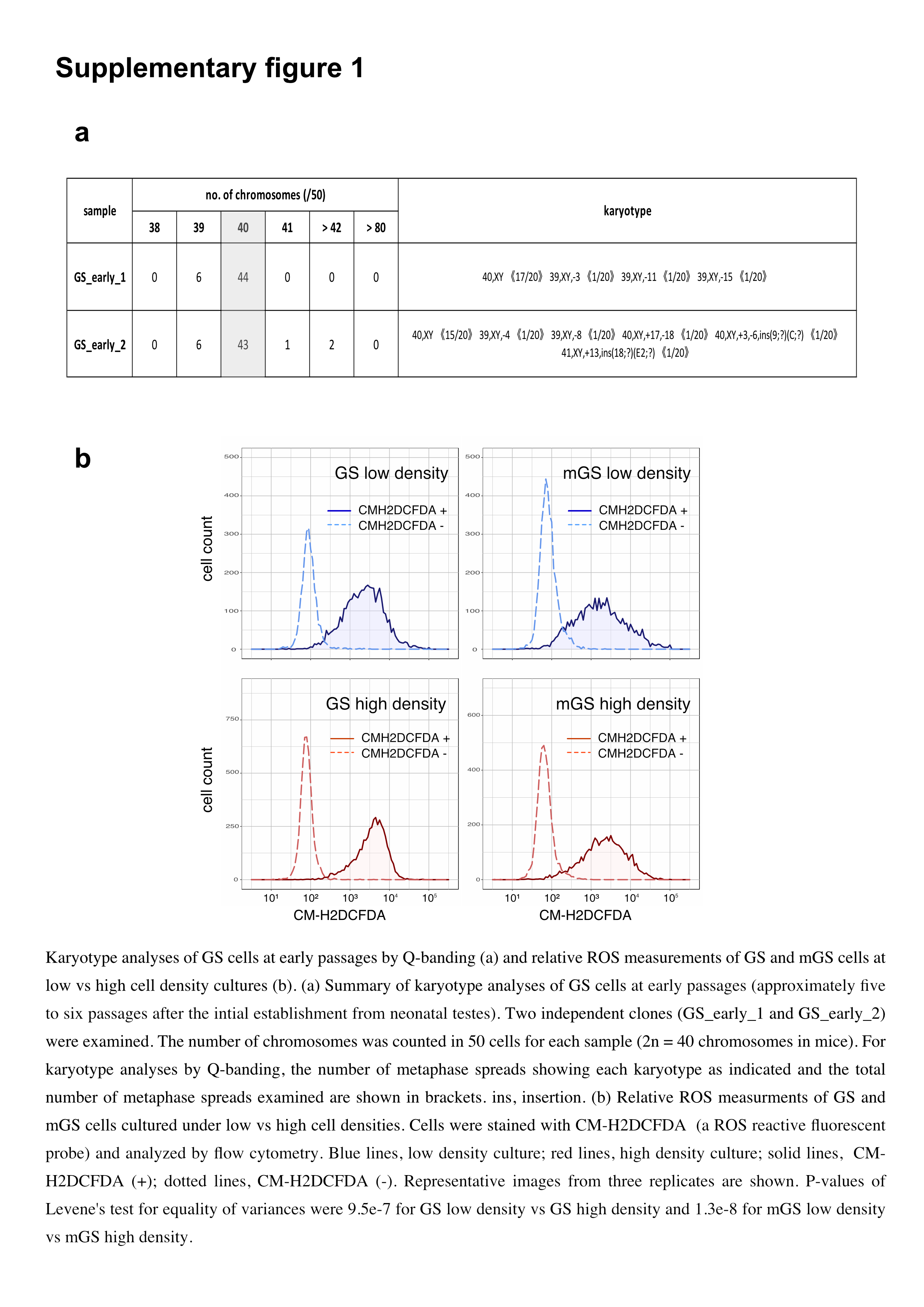

Supplement: Supplementary file 1 — Supplementary Figure 1. [file 41598_2021_3658_MOESM1_ESM.tiff]
